# Association between electronic cigarette use and respiratory outcomes among people with no established smoking history: a comprehensive review and critical appraisal

**DOI:** 10.1007/s11739-025-03894-7

**Published:** 2025-02-24

**Authors:** Arielle Selya, Giusy Rita Maria La Rosa, Lucia Spicuzza, Jaymin B. Morjaria, Grazia Caci, Riccardo Polosa

**Affiliations:** 1 Pinney Associates, Inc., 201 N. Craig St. Suite 320, Pittsburgh, PA 15213 USA; 2 Center of Excellence for the Acceleration of HArm Reduction (CoEHAR), University of Catania, Via S. Sofia, 78-Ed. 4, P. 2, 95123 Catania, Italy; 3 Department of Clinical and Experimental Medicine, University of Catania, Catania, Italy; 4 Respiratory Unit, University Teaching Hospital “Policlinico-S.Marco”, University of Catania, Catania, Italy; 5 Department of Respiratory Medicine, Harefield Hospital, Guy’s & St Thomas’ NHS Foundation Trust, Harefield, UK; 6 UOC MCAU, University Teaching Hospital “Policlinico-S. Marco”, University of Catania, Catania, Italy; 7 Institute of Internal Medicine, University Teaching Hospital “Policlinico-S.Marco”, University of Catania, Catania, Italy; 8 Department of Medicine and Surgery, “Kore” University of Enna, Enna, Italy

**Keywords:** Asthma, Chronic obstructive pulmonary disease, Electronic cigarettes, Never-smoking, Respiratory illness, Respiratory symptoms

## Abstract

Nicotine consumption in many countries is shifting away from combustible cigarettes and toward electronic cigarettes (ECs). Understanding the overall population-level impact requires weighing their possible benefits (e.g., for smoking cessation/switching) vs harms (e.g., long-term health risks). However, current evidence on health risks is limited by the absence of long-term data and confounding by prior cigarette smoking history. Focusing on short- to medium-term respiratory outcomes associated with EC use among people who never smoked (PWNS) is informative. We perform a narrative review and critical appraisal of studies examining the prospective association between exclusive EC use and respiratory outcomes among PWNS (either true never-smoking or never-established smoking). We included 12 studies with prospective designs that examine a range of respiratory outcomes subsequent to EC use among PWNS. Eight studies did not find statistically significant differences in respiratory risk associated with baseline EC use. The remaining five studies reported a significant association in at least one analysis, but in four of these studies, associations were not robust across models. Limitations included overreliance on data from the U.S. Population Assessment of Tobacco and Health, uncertain directionality (i.e., pre-existing respiratory conditions were not always ruled out), confounding by other combustible tobacco use, and small sample sizes. All but one study lacked clear and statistically significant evidence of self-reported respiratory *diagnoses* associated with EC use among PWNS, or showed a tenuous association with mild respiratory *symptoms*. This has favorable implications for ECs’ population health impact; however, small sample sizes and statistical biases limit this evidence. A formal systematic review on this topic is forthcoming.

## Introduction

Combustible cigarette smoking remains the primary cause of preventable premature death in many countries worldwide. Electronic cigarettes (ECs) are a non-combustible nicotine product and as such are substantially less harmful than cigarettes [1–3]. Given that ECs are at the lower end of the risk continuum [3–6] and are effective smoking cessation aids [7] among people who are actively trying to quit smoking in the near future—or alternatively, an appealing and lower-risk alternative consumer product for those who are *not* immediately planning to quit [8]—ECs have the potential to substantially reduce smoking-attributable mortality in the population if they displace cigarette smoking, according to simulation modeling studies [9–13].

**Fig. 1:**
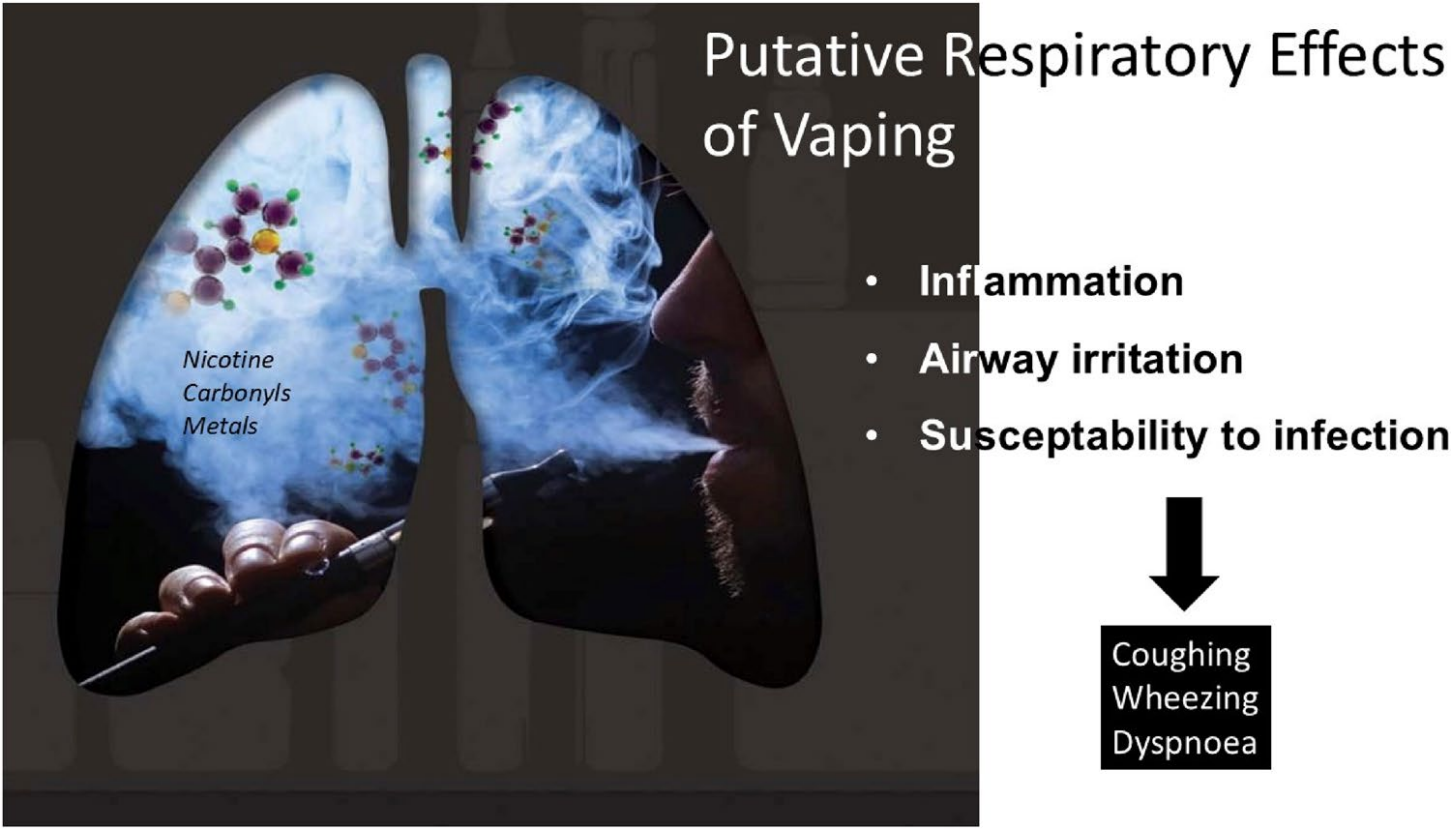
Possible mechanisms by which e-cigarettes could affect respiratory health

However, the ultimate population impacts of ECs also depend on their possible detrimental effects, especially from long-term and cumulative use. While ECs most likely pose substantially lower health risks *relative to* cigarettes, there may be some *absolute* level of risk from EC use alone (Fig. 1). Such absolute risks could attenuate some of the projected benefits of switching completely from cigarettes to ECs, as well as increase risks of EC use among people who smoke (i.e., dual use) and among people who never smoked (PWNS). We focus here on respiratory health outcomes, as e-cigarettes have been around for sufficiently long to theoretically impact respiratory health.

Current evidence on whether EC use uniquely poses measurable respiratory health risks has major limitations due to the nature of observational studies, which contain selection bias and confounding. In particular, since most people who use ECs have a history of smoking combustible cigarettes (either currently or formerly), any apparent association between EC use and a health outcome is likely confounded by smoking history. While many studies adjust for smoking status, this is often insufficient: adjusting for more detailed smoking history (e.g., using pack-years) is essential to account for the degree of smoking exposure [14, 15], yet most studies merely adjust for smoking status (current vs. former vs. never smoking; or even simply current vs. non-current smoking). The resulting residual confounding suggests that the *apparent* association between EC use and health outcomes could in reality be partly, or perhaps fully, explained by cumulative smoking history. Studies focusing on PWNS can avoid this confounding bias, and provide stronger evidence examining ECs’ possible direct health risks in humans; however, little is known about respiratory risks of EC use in this group, as EC use among PWNS is rare [16–18].

Here, we conduct a narrative review of existing literature on EC use by PWNS and respiratory outcomes, defined as broadly as possible. We focused on studies with prospective designs to avoid a common bias in cross-sectional studies on this topic (i.e., due to cases with the reverse temporal sequence where the respiratory outcome preceded EC use) [19]. We also critically appraise each study with respect to strengths and weaknesses and make recommendations for future research.

## Materials and methods

We performed a literature review to identify peer-reviewed studies that examined EC use and respiratory symptoms among PWNS and used a prospective study design. PubMed and Scopus databases were searched in April 2024 using the search terms: (“never-smokers” OR “never smokers” OR “never smok*” OR “näive” OR “healthy”) AND (“e-cig*” OR “e-cigarette” OR ENDS OR “electronic cigarette*”) AND (“respir*” OR “lung”) AND (“cohort” or “observational” or “follow-up” OR “randomized controlled trial” OR “RCT”). Additional manual searching was done in reference lists of included articles and in relevant peer-reviewed journals on either respiratory disease research or tobacco research.

All types of prospective study were included [e.g., clinical observational studies, randomized clinical trials (RCTs), and population surveys]. Cross-sectional studies were excluded due to uncertainty about the temporal sequence of exposure and outcome [19], as were laboratory studies, reviews, study protocols, case reports, conference abstracts, and articles not written in English.

Due to the small number of eligible studies, we retained studies regardless of their definition of never-smoking, i.e., either “true” never-smoking (i.e., never smoked even a puff in one’s lifetime) or never-*established* smoking (< 100 cigarettes/lifetime); see Limitations. Additionally, some studies were retained which analyzed people who currently or formerly smoke but which analyzed data in a way that allowed an approximate estimation of the effect among PWNS.

First, titles and abstracts of all search results were screened independently by two reviewers (GRMLR and GC), and eligible or potentially eligible articles were reviewed in full by three reviewers (AS, GRMLR, and GC) to determine final eligibility. Disagreements were resolved through discussion, bringing in a fourth reviewer (AS, GRMLR, GC, and RP). Some articles that lacked one of the criteria were retained for discussion on a case-by-case basis if all reviewers agreed they were nevertheless informative.

Critical appraisal was conducted through detailed reading of the full text and focused on the following considerations: (1) possible violations of the correct temporal sequence, e.g., if respiratory symptoms or conditions could have been present from baseline and therefore co-occurred or preceded EC use; (2) whether all relevant confounding factors were included in the model (e.g., other non-cigarette combustible tobacco use); (3) possible sample size limitations; (4) presence of sensitivity/supplementary analyses, and robustness of findings across different analyses; (5) plausibility of results in the model (e.g., whether available results align with a dose–response effect and are larger for current EC use than former-EC use); (6) how PWNS were handled in the model (i.e., subgroup analysis or adjustment for smoking status).

## Results

After applying inclusion and exclusion criteria, a total of 12 studies were included (see Table 1; see Table 2 for excluded studies). In this section, we provide only basic characteristics and main findings of each study, and in Discussion provide a more thorough summary of each paper along with an integrated critical appraisal of each study.

**Table 1:** Included studies and their characteristics

Article	Age group	Data source	No. of PWNS	Exposure/control group	Outcome	Significant association among PWNS?
Karey et al. [25]	Adults (18 +)	PATH, Waves 4–5	65	Current vs. former-EC use vs. never-EC use	Respiratory symptoms (functionally important: cutoff of 2 on 0–9 scale; 7 symptoms)	**No;** “Among never combustible tobacco smokers, no significant association was detected between e-cigarette use and important respiratory symptoms”
Reddy et al. [26]	Youth (12–17), Adults (18 +)	PATH, Waves 3–4		Current (some days or everyday) EC use vs. non-current use	Incidence/onset of self-reported respiratory symptoms in past year	**No;** non-significant due to confidence interval containing 1.00: “exclusive [EC] users (adjusted odds ratio [AOR] vs. noncurrent users, 1.17; 95% CI, 0.79–1.74)”
Sargent et al. [14]	Young adults (18–24)	PATH, Waves 1–3		Current (some days or everyday) EC use vs. non-current use	Respiratory symptoms (functionally important: cutoff of 3 on 0–9 scale; 7 symptoms)	**Sometimes;** prevalence outcome: “Compared to never users, the risk of functionally important respiratory symptoms were not significantly different for exclusive users of e-cigarette” worsening outcome: “…findings for exclusive e-cigarette use were sensitive to symptom severity, showing a significant association with worsening symptoms at a threshold of ≥ 2… but not at a symptom threshold of ≥ 3” improvement outcome: “…e-cigarette users at a threshold of ≥ 3… were also more likely [to] show symptom improvement compared to never users” *[Note: these comparisons were not solely among PWNS and may be biased; see Discussion]*
Kenkel et al. [28]	Adults (18 +)	PATH, Waves 1–3	12	Current (some days or everyday) EC use and used ECs fairly regularly (vs. non-current or never regular)	Self-reported diagnoses of respiratory disease (COPD, chronic bronchitis, emphysema, or asthma)	**No;** “…among respondents who had never smoked combustible tobacco, we find no evidence that current or former e-cigarette use is associated with respiratory disease”
Stevens et al. [27]	Youth (12–17)	PATH, Waves 3–4	2998	Current vs. former-EC use vs. never-EC use	Respiratory symptoms (functionally important: cutoff of 2 on 0–9 scale; 7 symptoms)	**No;** “Baseline e-cigarette use did not increase the odds of having functionally important respiratory symptoms at follow-up regardless of combustible tobacco use status”
Polosa et al. [29]	Adults (18 +)	Bespoke sample of Italian adults, 3.5-year follow-up	9	Daily EC use of 3 + months vs. never-EC use	Lung function, respiratory symptoms, eNO, eCO, and HRCT of lungs	**No;** “this study did not demonstrate any health concerns associated with long-term use of EC in relatively young users who did not also smoke tobacco”
Sanchez-Romero et al. [23]	Adults (18 +)	PATH, Waves 1–5	51	Current (some days or everyday) EC use vs. non-current use	Self-reported wheezing in past 12 months	**No;** “Associations were small and not statistically significant for the odds of self-reported wheezing among never cigarette and current ENDS use when compared with never cigarette and noncurrent [EC] use”
Polosa et al. [30]	Youth (US middle and high school students)	Review and commentary of US youth survey results and respiratory risks				**Sometimes;** “Although vaping has been linked to respiratory symptoms, they tend to be transient and of uncertain significance”
Patel et al. [20]	Youth (12–17)	PATH, Waves 1–5	142	P30D EC use vs. no P30D use	Incidence/onset of self-reported asthma diagnosis	**No;** “…adolescents using [EC] exclusively… did not [have a statistically significant higher risk of incident diagnosed asthma at follow-up]”
Perez et al. [21]	Youth (12–17), Adults (18 +)	PATH, Waves 1–6	96 youth 160 adults	P30D EC use vs. no P30D use	Age of asthma onset	**Sometimes;** main analysis: “adults who reported never using cigarettes… and P30D ENDS use at the first wave of participation had increased risk of asthma incidence at earlier ages in comparison to adults who reported no P30D [EC] use” Supplementary analysis: Non-significant due to confidence interval containing 1.00: (never use of combustible TP and P30D [EC] use (vs. never use of combustible TP and no P30D [EC] use), AHR = 0.93 (0.70–1.22))
To et al. [22]	Adults (15–60)	CCHS with linkage to administrative health records	75	Current vs. non-current EC use	Asthma attack in past 12 months, among those with asthma	**Sometimes;** asthma prevalence outcome: “EC users had increased odds of prevalent asthma compared with nonusers… but the association was not statistically significant” Asthma attacks outcome: “EC use… showed significant interaction with sex… Female EC users and nonusers had a significant twofold increase in odds of asthma attacks compared with male nonusers” *[Note: these comparisons were not solely among PWNS and may be biased; see Discussion]*
Xie et al. [24]	Young adults (18–24)	PATH, Waves 1–5	312	Current vs. former-EC use vs. never-EC use	Onset of respiratory symptoms (wheezing)	**Yes;** “Current e-cigarette use was associated with higher odds for any respiratory symptom… and wheezing in the chest… Associations persisted among participants who never smoked combustible cigarettes”

*CCHS* Canadian Community Health Survey, *COPD* chronic obstructive pulmonary disease, *EC* electronic cigarette, *eCO* expired carbon monoxide, *eNO* expired nitric oxide, *HRCT* high-resolution computed tomography, *P30D* past 30-day, *PATH* Population Assessment of Tobacco and Health, *PWNS* people who never smoked (i.e., < 100 cigarettes/lifetime, including “not even a puff”), *TP* tobacco products. Bold text: overall conclusion about the presence of a significant association. Underlined text: subheadings for specific analyses, if applicable

**Table 2:** Excluded studies and reason for exclusion

Article	Truly longitudinal?	Investigates never-smoking individuals?
Zavala-Arciniega et al. 2024, *Res Square* https://doi.org/10.21203/rs.3.rs-3793149/v1	YES	NO^a^
Delmas et al. 2024, *Respir Med* https://doi.org/10.1016/j.rmed.2023.107496	NO	YES
Mukerjee et al. 2024, *Am J Prev Med* https://doi.org/10.1016/j.amepre.2023.12.005	YES	NO^a^
Cheney et al. 2023, *Prev Med Rep* https://doi.org/10.1016/j.pmedr.2023.102473	NO^b^	NO^a^
Tackett et al. 2024, *Thorax* https://doi.org/10.1136/thorax-2022-218670	YES	NO^a^
Mattingly et al. 2023, *Prev Med* 10.1016/j.ypmed.2023.107512	YES	NO^a^
Chaiton et al. 2024, *Tob Induc Dis* https://doi.org/10.18332/tid/156839	NO	YES
Berlowitz et al. 2023, *Am J Prev Med* https://doi.org/10.1016/j.amepre.2022.10.006	YES	NO^a^
Cordova et al. 2022, *Prev Med Rep* https://doi.org/10.1016/j.pmedr.2022.102016	YES	NO^a^
Dai et al. 2020, *NTR* https://doi.org/10.1093/ntr/ntaa180	YES	NO^a^
Tackett et al. 2020, *JAMA Netw Open* https://doi.org/10.1001/jamanetworkopen.2020.20671	YES	NO^a^
Bhatta and Glantz 2020, *Am J Prev Med* https://doi.org/10.1016/j.amepre.2019.07.028	YES	NO^a^

^a^Study only examined current vs. non-current smoking and thus combines former and never-smokers

^b^While some of the analyses were truly longitudinal, they were in the reverse direction of the research question (i.e., asthma predicting later cigarette and e-cigarette use)

Respiratory outcomes varied across studies: three studies analyzed self-reported asthma (incidence/onset of self-reported asthma in Patel et al. [20], age of self-reported new onset in Perez et al. [21], and self-reported prevalence and past-year asthma attacks in To et al. [22]); two studies analyzed wheezing symptoms (self-reported past-year wheezing in Sanchez-Romero et al. [23] and onset of self-reported wheezing symptoms in Xie et al. [24]); four studies analyzed an index of self-reported respiratory symptoms, some which used cut-off values denoting functionally important symptoms (Karey et al. [25]; Reddy et al. [26]; Stevens et al. [27]; Sargent et al. [14]); one study analyzed *any* self-reported respiratory diagnoses (COPD, chronic bronchitis, emphysema, or asthma; Kenkel et al. [28]); one study analyzed lung function (using expired biomarkers, spirometry tests, and high-resolution computed tomography; Polosa et al. [29]); and one study was a review and commentary on youth EC use (Polosa et al. [30]). Seven studies focused on the general adult population, two focused on young adults (ages 18–24), and five (some of which also examined adults) focused on youth. A total of 5 studies were identified for adults and 2 for young people. Table 3 presents the aggregate findings for each of the above outcome categories, along with notable limitations (discussed in detail in Discussion).

**Table 3:** Overview of aggregate results by type of outcome and notable limitations

Type of outcome	Studies	Aggregate findings (see Table 1 for detailed findings)	Limitations (see Discussion for details)
Self-reported asthma outcomes	Patel et al. [20] Perez et al. [21] To et al. [22]	No evidence for association with asthma incidence/onset [20]; tenuous association with age of asthma onset [21] (see Limitations); No evidence for association with asthma prevalence or past-year asthma attacks overall Annals ATS [39], but possible interaction with sex for past-year asthma attacks [22] (see Limitations)	In Perez et al. [21], tenuous association with age of asthma onset may be due to residual confounding by other combustible tobacco use; N.S. after accounting for this In To et al. [22], interaction with sex may be entirely due to sex; no clear evidence of a unique association with EC use
Wheezing symptoms	Sanchez-Romero et al. [23] Xie et al. [24]	No evidence association with self-reported past-year wheezing [23]; association with onset of wheezing [24]	Xie et al. [24] have possible confounding by other combustible tobacco use, which was not accounted for
Respiratory symptom index	Karey et al. [25] Reddy et al. [26] Stevens et al. [27] Sargent et al. [14]	No evidence for association with prevalence of functionally important symptoms [14, 25, 26]; tenuous association with both worsening and improvement of functionally important symptoms depending on cut-off value [14]	Reddy et al. [26] did not account for prior smoking history and associations could be due to cumulative smoking history In Karey et al. [25], stronger association among former (vs current EC use) is not plausible, suggesting unaccounted-for confounding Sargent et al. [14] adjusted for smoking status rather than analyzing PWNS separately, which may introduce bias In all studies here, small numbers of PWNS who used ECs may limit statistical power
*Any* self-reported respiratory diagnosis	Kenkel et al. [28]	No evidence for association with prevalence of any self-reported respiratory diagnosis	Small sample size of PWNS who used ECs (*n* = 12) prevented reliable estimates
Lung function, eNO, eCO, and HRCT of lungs	Polosa et al. [29]	No evidence for effects on lung function	Small sample size of PWNS who used ECs (*n* = 9) prevented statistical analysis
Review and commentary on youth EC use	Polosa et al. [30]	Some evidence for transient respiratory symptoms of uncertain clinical significance	Reflects limitations of underlying studies, e.g., unaccounted-for confounding

*EC* electronic cigarette, *eCO* expired carbon monoxide, eNO expired nitric oxide, *HRCT* high-resolution computed tomography, *P30D* past 30-day, *PATH* Population Assessment of Tobacco and Health, *PWNS* people who never smoked (i.e., < 100 cigarettes/lifetime, including “not even a puff”), *TP* tobacco products

Seven studies reported no significant association between baseline EC use and subsequent respiratory outcomes among PWNS. Five reported at least one significant association, but in four of these studies, this result was not robust across different models presented (see Table 1). The majority of studies (n = 9) analyzed data from the Population Assessment of Tobacco and Health (PATH), a nationally representative longitudinal US survey of youth and adults. With one exception (Polosa et al. [29]; see Discussion), none of the studies provided detailed EC device characteristics (e.g., nicotine concentration, flavor, or device type).

## Discussion

We identified 12 relevant studies examining the association between EC use and subsequent respiratory outcomes among PWNS. Here, we discuss each paper’s findings in detail and critically appraise the strengths and weaknesses of each study, organized first by main finding (whether or not a significant association between EC use and respiratory outcome was found in at least one study) and next by age group.

### Studies reporting no association: summary and critical appraisal

#### Adults

In adults, a 2-year study by Karey et al. [25] and a similar timeframe study by Reddy et al. [26] both found no significant association between baseline EC use and the development of functionally important respiratory symptoms (using a 7-item index of symptoms, with a cutoff of a mean score of 2+ on a 0–9 scale) in adults who never smoked. Both studies analyzed consecutive waves of the longitudinal Population Assessment on Tobacco and Health (PATH) survey in adults.

A limitation of existing (predominantly cross-sectional) studies of e-cigarette use and health outcomes is bias due to the presence of participants whose respiratory symptoms preceded EC use [19]. To overcome this limitation, both Karey et al. [25] and Reddy et al. [26] excluded participants who already had a diagnosis of respiratory disease [25] or respiratory symptoms [26] at the baseline wave. This is a notable strength of both studies as it ensures the correct temporal sequence to examine whether e-cigarettes have a causal effect on the *development* of respiratory symptoms. An additional strength of Karey et al. [25] was to stratify by cigarette status (current, former, or never) which allowed an examination of respiratory symptoms uniquely associated with e-cigarette use (i.e., among never-smokers, whose respiratory symptoms cannot be attributed to smoking history). Reddy et al. [26] on the other hand, only examined *current* e-cigarette use, without accounting for prior smoking history: had there been a significant association between e-cigarette use and onset of respiratory symptoms, it could be due to prior cigarette smoking, in which case adjusting for pack-years would be necessary (though not necessarily sufficient) to account for this [14].

Limitations still apply to both Karey et al. [25] and Reddy et al. [26] due to the nature of observational data. Specifically, there may be spurious associations due to other factors that were unadjusted for: for example, Karey et al. [25] showed that among adults who *formerly* smoked, former EC, but not current EC use, was associated with higher odds of developing respiratory symptoms. Since it is not biologically plausible that former-EC use could have a causal effect, while current use does not, this likely indicates the influence of additional confounding factors. Additionally, since there were few never-smoking adults who used ECs, the ability to detect significant associations with respiratory symptoms may have been limited, calling for future research to seek out larger samples of adults who use ECs but never-smoked cigarettes. Overall, nevertheless, these findings lack clear evidence for moderate or severe respiratory risks of EC use over the medium term among never-smoking adults.

Similarly, Sargent et al. [14] analyzed adult PATH participants without a diagnosis of COPD at the baseline wave and found no significant differences in the *prevalence* of respiratory symptoms at the follow-up wave between adults who exclusively used ECs vs. did not use ECs at baseline. (This same study did, however, find significant associations between EC use and *changes* in respiratory symptoms—both worsening and improvement; see section below.) Similarly to the studies above, a notable strength of this study is including only participants *without* baseline diagnoses of respiratory disease, ensuring the correct temporal sequence for examining the possible effects of e-cigarette use. A limitation of this analysis [14] is that Sargent et al. did not run a dedicated analysis containing solely never-smoking adults, but rather statistically adjusted for smoking status (i.e., had smoking status as a control variable, using never-smoking as the reference group). In the context of a multivariate regression, each estimate is interpreted as the change in that variable *with all other variables in the model held constant*: i.e., the odds ratio for EC use should reflect the effect of exclusive EC use regardless of whether a participant never, formerly, or currently smoked (provided that all model assumptions are met). However, there still may be some bias (toward a positive association) from including all smoking status groups in the same model, especially if there is an unaccounted-for interactive effect between EC use and smoking history (such that those with longer smoking histories have higher health risks) which could falsely appear to carry over to PWNS.

A study by Kenkel et al. [28] conducted a replication and extension of a prior cross-sectional study by Bhatta and Glantz [31], the latter of which reported that EC use was associated with significantly higher odds of developing respiratory symptoms among adults in PATH, despite excluding those who already had respiratory disease at baseline. However, Kenkel et al. [28] noted that in Bhatta and Glantz’s analysis [31], the majority of EC users currently or formerly smoked: only 12 participants out of 17,601 used ECs but had never smoked cigarettes, and none of these 12 developed respiratory symptoms in the PATH waves examined. Since this group was too small for a formal statistical analysis, Kenkel et al. [28] instead replicated Bhatta and Glantz’s analysis [31] but examined categories of e-cigarette and cigarette use. Results showed no evidence of respiratory disease over a 3-year period in never-smoking adults who used ECs. Further, among adults who formerly and currently smoked, there was no marginal independent association between EC use and respiratory outcomes over and above smoking status, which is consistent with Sargent et al.’s conclusion [14] that cigarette smoking largely explains onset of respiratory symptoms, with no additional risk introduced by ECs. Limitations also apply to Kenkel et al.’s analysis, however, due to the observational nature of the data and the small sample size of adults who used ECs but never smoked (N = 12) [28]. These studies collectively indicate that while some individuals who use ECs may experience mild respiratory symptoms, evidence is lacking for an overall medium-term impact of ECs on lung health in the absence of an established smoking history.

Stevens et al. [27] examined e-cigarette use and respiratory symptoms among youth in PATH Waves 3–4. They focused on youth without asthma at baseline, which is a strength of the study as it excludes youth who may have pre-existing respiratory symptoms due to asthma. Stevens et al. [27] found that youth who exclusively used ECs at baseline did not have significantly higher odds of reporting functionally important (cutoff of 2 on a 0–9 scale) respiratory symptoms (7-item scale related to wheezing). This was true regardless of combustible tobacco history (i.e., there was no significant association specifically among never-smoking youth).

Similarly, a 3.5-year prospective observational study of daily ECs’ users without a history of smoking by Polosa et al. [29] found no significant alterations in lung function, respiratory symptoms, or exhaled breath nitric oxide (eNO), and no structural abnormalities in high-resolution computed tomography (HRCT) scans. However, the study faced limitations, such as a small sample size, which diminished its power to detect abnormalities or significant changes over time, the potential for selection bias (as the study may have disproportionately included healthier e-cigarette users), and a medium-term follow-up duration. This was the only study in the current review to collect EC device characteristics; 6/9 EC users used e-liquid containing nicotine (0.9–1.8% strength), with the strength declining over the course of the study; most (7/9) used tobacco-flavored e-liquid; and there was a range of device type (advanced or standard refillable).

Sanchez-Romero et al. [23] analyzed wheezing symptoms specifically in the longitudinal PATH survey, and found that never-smoking adults who used ECs at baseline had no increase in subsequent wheezing risk over a 5-year period in the US. In contrast, adults who exclusively smoked or dual used at baseline had significantly higher odds of subsequently developing wheezing symptoms, as did adults who formerly smoked and used ECs. However, these associations are likely attributable to cigarette smoking history rather than EC use, considering that there was no risk among never-smoking adults who exclusively used ECs.

#### Youth and young adults

A review of EC use in young people also showed no clinically significant lung symptoms or asthma triggers that are uniquely attributable to e-cigarette use [30]. Instead, the previous reports of significant respiratory symptoms in young people who use ECs are more likely explained by priori *cigarette* smoking history, consistent with the conclusions of Sargent et al. [14] and Kenkel et al. [28] in adults. Alternatively, the association could be due to confounding with the other conditions that may have preceded EC initiation [19]—especially in the case of outcomes related to asthma, which is often diagnosed in childhood [30]. The only respiratory symptom uniquely and consistently associated with EC use in this review is cough, but this may be a transient effect of respiratory irritation, and does not seem to progress into clinically significant lung disease [30].

Several other studies showed that youth who exclusively use ECs, unlike those who exclusively use cigarettes or dual use, are no more likely than non-users to develop asthma, including Patel et al. [20] and Perez et al. [21]. Like above, these two analyses focused on youth without an asthma diagnosis at baseline, to rule out cases where asthma preceded EC use. Similar to the studies on adults, any association with respiratory conditions seems to be primarily driven by cigarette smoking, with no significant evidence that exclusive EC use is associated with developing asthma.

### Studies reporting a significant association: summary and critical appraisal

Despite the studies above being reassuring in lacking statistically significant evidence of respiratory outcomes of EC use by both youth and adults who never smoked, other studies have reported mixed outcomes indicating possible respiratory risks following EC use. For example, in an analysis of PATH longitudinal data among both youth and adults, Perez et al. [21] reported that among adults who never smoked at baseline and did not have asthma or COPD, those who used ECs at baseline were significantly more likely to subsequently develop asthma at an earlier age than those who did not use EC. (Perez et al. did not find a significant association among *youth,* but imply that there could be a true association that was not detected due to low statistical power [21]). However, it is likely that this apparent association between baseline EC use and earlier age of asthma onset among adults is nevertheless confounded by combustible tobacco use: not only did Perez et al. [21] include participants who used other tobacco products at baseline, but they included participants who subsequently initiated cigarette smoking *after* baseline. In fact, Perez et al.’s own supplementary analyses showed that when subsetting further to participants who never-smoked cigarettes *and* did not use other tobacco products, baseline EC use was no longer significantly associated with age of asthma onset [21]. Therefore, though Perez et al. conclude that EC use is associated with age of asthma onset [21], in our judgment, this may be a false-positive due to unaccounted-for confounding by other combustible tobacco use.

To et al. [22] published another study reporting a possible link between EC use and asthma outcomes, using the Canadian Community Health Survey with linkage to health administrative data at a later timepoint. Analyses showed that EC use was not significantly associated with *prevalence* of asthma, nor (as clarified by a recent erratum) past-year asthma attacks among participants with asthma, but Annals ATS [39] there was a significant interaction between EC use and sex in the analysis of past-year asthma attacks among participants with asthma (OR = 2.30, 95% CI 1.29–4.12 for females who used ECs vs. males who did not). However, this association appears to be entirely explained by the sex difference alone [i.e., the comparison of female vs. male EC users showed an identical association (OR = 2.29, 95% CI 1.57–3.35)] as pointed out by post-publication criticism [40]. While it is possible that EC use may be associated with asthma *control* among adults who already have asthma, we consider this to be an unreliable result due to confounding by sex. Finally, like Sargent et al. [14], To et al. [22] adjusted for smoking status (i.e., by including it as a covariate, with never-smoking as the reference group) rather than conducting a dedicated analysis to PWNS. While the estimates of EC use should approximately apply to never-smoking adults, there is the possibility that including formerly and currently smoking adults in the model could bias the results toward the positive.

While Sargent et al. [14] as noted above did not find EC use to be significantly associated with *prevalence* of functionally important respiratory symptoms (as noted in the section above), they did find exclusive EC use to be associated with *worsening* of functionally important respiratory symptoms in some analyses, though it depended on the exact cutoff used to denote “functionally-important” (i.e., 2 vs 3 on a 9-point scale) [14]. However, an opposing association was also found: exclusive EC use was associated with significantly higher odds of symptom improvement when using the higher cutoff (i.e., 3). It is not clear how to reconcile these apparently contradictory findings, but if they reflect true causal associations, it is possible that EC use could have opposing effects for different groups of people, possibly depending on their initial symptom severity. However, we note again the possible bias from including adults who formerly and currently smoked in the analysis, rather than conducting a focused analysis of only PWNS. For example, EC use by PWNS could explain why EC use is associated with *worsening* symptoms, while EC use by people who switched away from smoking could explain why EC use is also associated with symptom *improvement*.

Finally, Xie et al. analyzed the onset of respiratory symptoms among young adults (ages 18–24) without respiratory disease or respiratory symptoms at baseline in PATH Waves 2–5. Analyses stratified the associations between EC and respiratory symptoms by combustible smoking status and showed that EC use was associated with significantly higher odds of developing respiratory symptoms among all smoking history groups: though the odds ratios of EC use were attenuated among never-smoking youth, they remained statistically significant. Notably, this is the only study of the 12 identified that reported a significant association that was robust across models. This may reflect a true association between EC use and coughing/wheezing; however, there is still possible confounding by other combustible tobacco use. While the analyses adjusted for the use of other tobacco products including cigars, cigarillos, and other combustible tobacco, the adjustment was done based on current use, and did not distinguish between former and ever use of other combustible tobacco products. Thus, it is not clear whether, or to what extent, the association between EC use and onset of coughing/wheezing may be due to prior use of combustible tobacco. However, the odds ratios associated with these were modest compared to that of EC use, suggesting that EC use may pose increased risk for coughing and wheezing among never-smoking young adults.

### Limitations

Existing evidence is sparse, likely owing to the low prevalence of EC use among PWNS. As a result, we included a range of respiratory outcomes, and evidence on a given respiratory disease was limited. Similarly, we included different definitions of “never-smoking,” but never-*established use* can include those with nontrivial levels of tobacco use and experimentation [32, 33], which could confound the association between EC use and respiratory outcomes. More generally, as these are all observational studies, causality cannot be established, and there may be unaccounted-for confounding. The non-significant findings also indicate the absence of evidence, as none of these studies assessed evidence of absence. Collectively, the studies were primarily US-centric, as many used the same PATH dataset. Finally, no studies examined detailed measures of EC use (frequency, duration, or device characteristics).

### Summary and implications

Overall, the 12 studies in this narrative review show, at best, a lack of evidence for a statistically significant association between EC use and respiratory risks, and at worst, an association with mild respiratory symptoms (coughing or wheezing). Of the five studies that did report at least one significant association, four showed the association to be tenuous and variable across different models, while only one showed it to be robust. In some studies presenting variable findings, the analyses showing a positive association may be biased (i.e., false positive) due to unaccounted-for confounding [21, 22], while others suggest that the association might depend on how strictly the outcome is defined (i.e., cutoff for functional importance) [14]. Collectively, these findings suggest that ECs may pose some mild respiratory risks, but it is unclear whether this is clinically significant. In comparison to combustible cigarettes, ECs are therefore likely to pose much lower respiratory risks. However, it is important to keep in mind key limitations, namely that the evidence available is primarily specific to the US population and may not generalize to other populations, and that the small sample size of PWNS who use ECs introduces uncertainty into these results and may fail to detect some significant associations.

While this narrative review focuses on PWNS, these findings—thought they are tenuous and based on limited evidence—could tentatively generalize to other groups, including people who currently and formerly smoke, as they estimate potential unique effects of exclusive EC use. For example, people who currently smoke are already facing much more severe respiratory risks from cigarette smoking, and the possible harms of adopting ECs (i.e., dual or even synergistic exposures) may be outweighed by the possible benefits (i.e., discontinuing EC use entirely [7], or substantially reducing cigarette consumption [34]). Additionally, former smokers who switched away from smoking and now exclusively use ECs are likely to experience a net benefit, having moved from a product that poses severe respiratory risks to one that may pose only mild risks. On the other hand, PWNS and use ECs may experience a net risk. However, this depends on the counterfactual of what the person would have done otherwise: if they would have remained nicotine-naïve, this represents a net harm that must be factored in to the total population health impact; but if they would have smoked cigarettes and were diverted by ECs [35], they may experience a similar benefit as former smokers. These competing pathways and possible costs vs. benefits must be continuously re-evaluated as new evidence on possible health risks of EC emerges.

### Recommendations for future research

Together, the above narrative review identified relatively few studies that have focused on PWNS to examine the question of whether EC use itself poses respiratory health risks. This is an under-studied population which is especially informative for this question, as focusing on this population avoids confounding by cigarette smoking history that plagues most research on this question.

This evidence gap motivates more rigorous research on this question, using prospective studies and larger samples of PWNS. Since current samples have low prevalence of EC use among PWNS, some of the existing studies reviewed here may have insufficient statistical power, particularly when thoroughly adjusting for confounding factors, and may have therefore missed true significant associations. Thus, future research may benefit from dedicated samples of PWNS who use ECs, rather than relying on the small prevalence of this group in more general samples. Provided sufficient sample sizes can be obtained, future research should also more thoroughly adjust for confounding factors and pre-existing conditions that could influence the association. The body of research would also be strengthened by studies focusing on trajectories of symptoms or disease severity over time among participants with respiratory conditions. Finally, given that several papers in this review reported a tenuous or ambiguous association whose significance depended on model specification, we recommend that future papers present several sensitivity analyses to allow an evaluation of robustness.

As an immediate next step, a sister publication [36] will pursue a formal systematic review that draws on the insights obtained by the current narrative review and critical appraisal. Additionally, we have recently published our findings from a dedicated sample of never-smoking adults on this topic: the Vaping Effects: Real-World International Surveillance (VERITAS) Study [37], a global sample of adults without a history of established smoking. Our recent analysis of respiratory symptoms showed that the vast majority of both the vaping and control cohorts “rarely” or “never” experienced respiratory symptoms; those who vaped had statistically significantly – but not clinically significantly – more frequent respiratory symptoms [38]. These findings align with the current findings, but we plan to continue this work to examine longer follow-up periods. The VERITAS project will substantially add to the existing evidence by comparing respiratory symptoms among a large sample of never-smoking adults who use vs. do not use ECs.

## Conclusions

We provide a narrative review documenting the evidence assessing possible respiratory risks associated uniquely with EC use among PWNS. Overall, evidence is lacking for moderate-to-severe respiratory risks associated with EC use, but there was a tenuous association with mild symptoms that was not robust across models and may be due, in part, to unaccounted-for confounding. While ECs are not completely risk-free, evidence to date suggests that shifting away from combustible tobacco toward ECs could have an overall positive impact on respiratory health of the population. This evidence also alleviates concerns about moderate-to-severe *absolute* respiratory risks associated with long-term EC use by people who switched completely away from smoking. Healthcare providers should encourage people who smoke and are unlikely to quit using other methods to switch completely to ECs and regulation should be risk-proportionate to incentivize people moving down the continuum of harm. However, this evidence is limited and imprecise and is over-reliant on US samples. Additional research on possible health harms associated with EC use by PWNS, especially with larger samples, longer follow-ups, and more thorough control for confounding factors, is essential for understanding the population health impacts of ECs.

## Data Availability

All data generated or analysed during this study are included in this published article and its supplementary information files.

## Abbreviations

AHR: Adjusted hazard rate
ASH: Action on Smoking and Health
AOR: Adjusted odds ratio
ARR: Adjusted risk ratio
CI: Confidence interval
COPD: Chronic obstructive pulmonary disease
EC: Electronic cigarette
eCO: Expired carbon monoxide
eNO: Expired nitric oxide
EPA: Environmental Protection Agency
HRCT: High-resolution computed tomography
NHIS: National Health Interview Survey
PATH: Population Assessment of Tobacco and Health
PICO: Population, intervention, comparator, outcomes
PRISMA: Preferred Reported Items for Systematic Reviews and Meta-Analyses
RCT: Randomized controlled trial
VERITAS Study: The Vaping Effects: Real-World International Surveillance Study
WHO: World Health Organization
